# Supplementary material for: Physiological Cell Culture Media Tune Mitochondrial Bioenergetics and Drug Sensitivity in Cancer Cell Models
Source: Cancers (Basel). 2022 Aug 13;14(16):3917. doi: 10.3390/cancers14163917 (PMC9405899; doi:10.3390/cancers14163917)
Supplement: Supplementary file 1 [file cancers-14-03917-s001.zip › cancers-1842678-supplementary.pdf]

cancers

MDPI

Supplementary Information for

This PDF file includes:

Figures S1 to S3
Tables S1 to S2

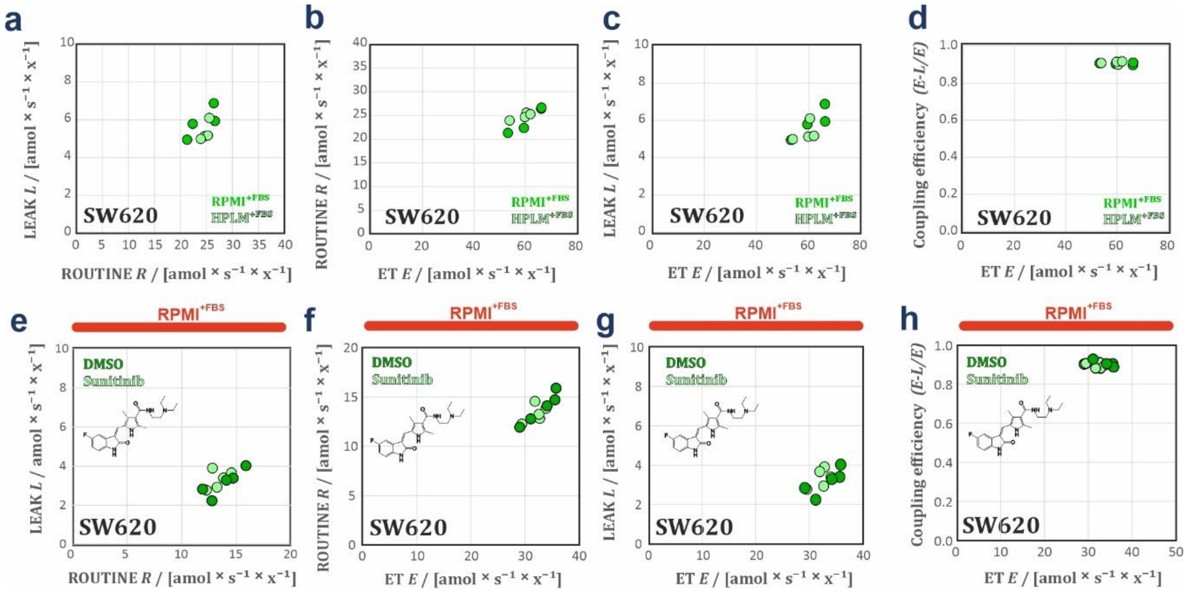

Figure S1. Bioenergetic cluster analysis in different respiratory coupling states of the SW620 cells in steady state and exposed to sunitinib grown in classical and HPLM media. BCA plots of steady state conditions **(a – d)** and sunitinib-exposed SW620 cells **(e - h)**. Plots of *L* over *R*, *R* over *E*, and *L* **(a - c),** and coupling efficiencies *(E-L)/E* **(d)** over ET capacities in SW620 cells showing no cluster differences between RPMI^{+FBS} and HPLM^{+FBS}. Plots of *L* over *R*, *R* over *E*, and *L* **(e - g)** and coupling efficiencies *(E-L)/E* over *E* **(h)** in SW620 cells grown in RPMI^{+FBS} and exposed to either DMSO or 10 μM sunitinib for 3 h. No cluster differences between DMSO and sunitinib are observed. Sunitinib structure is represented for drug-exposed SW620 cells **(e – h)**.

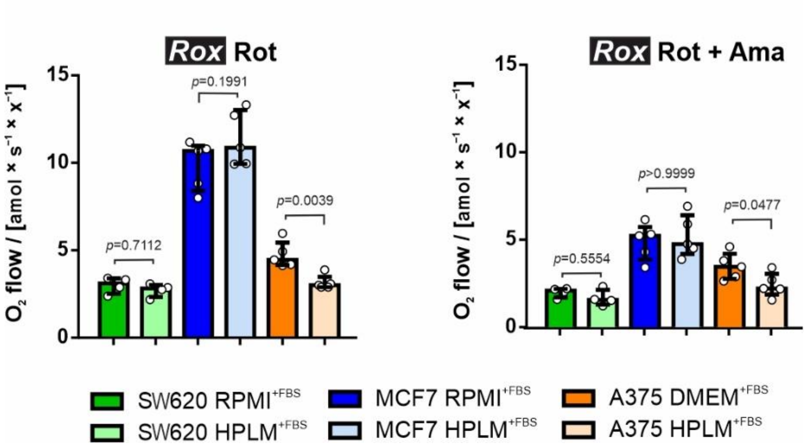

Figure S2. *Rox* in classical and HPLM+FBS media in SW620, MCF7, and A375 cell populations. Comparison of residual O_2 consumption (*Rox*) after rotenone (Rot, left panel) and rotenone + antimycin A additions (Rot + Ama, right panel) in SW620, MCF7 and A375 cell lines grown in RPMI+FBS / DMEM+FBS and HPLM+FBS. Values of O_2 flow [amol × s^{-1} × x^{-1}] are represented as median ± IQR (50% range). 2-wayANOVA with Bonferroni´s multiple comparison test; *N* = 4.

a

SW620 RPMI+FBS		SW620 HPLM+FBS	
DMSO	Sunitinib 10 µM 3h	DMSO	Sunitinib 10 µM 3h
99.50 % ± 0.21	99.63 % ± 0.16	99.31 % ± 0.11	99.36 % ± 0.22

b

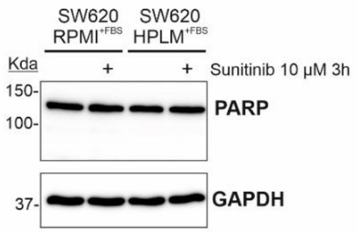

Figure S3. Sunitinib short-term treatment does not affect cell survival and apoptosis in SW620 cells. **(a)** Viability values of SW620 grown in in RPMI+FBS and HPLM+FBS and exposed to 10 µM sunitinib for 3 h. Viability was measured in living cells by the Casy® TT cell counter and analyser (Roche Innovatis AG, Germany). Values are median ± IQR (*N*=5) **(b)**. Western-Blot analysis of PARP-1 (46D11, Cell Signaling) of SW620 cells grown in RPMI+FBS and HPLM+FBS, and exposed to 10 µM sunitinib for 3 h. Sunitinib (+)/DMSO control. GAPDH (14C10, Cell Signaling) served as loading control (*N* = 4).

cancers

MDPI

Table S1. Respiration of cancer cell lines SW620, MCF7, and A375 in classical media (RPMI or DMEM) and HPLM. Median ± IQR (50% range). **(a)** *R*, *L* and *E* corrected for residual oxygen consumption *Rox*. *N* are biological replicates. **(b)** Flux control ratios, net capacities and flux control efficiencies normalized for ROUTINE or ET capacity as an internal normalization to express respiration independent of cell count. *Rox*/E'_{tot} ratio is *Rox* normalized against the total E'_{tot}. E'_{tot} is the ET capacity without *Rox* correction. Bold case indicates statistical significance at $p \leq 0.05$.

**a** cell line - culture media	number of repeats *N* or groups	ROUTINE *R* [amol×s^{-1}×x^{-1}]	LEAK *L* [amol×s^{-1}×x^{-1}]	ET capacity *E* [amol×s^{-1}×x^{-1}]	*Rox* [amol× s^{-1} × x^{-1}]
SW620-RPMI^{+FBS}	*N = 4*	24.39 ± 4.82	5.86 ± 0.66	62.83 ± 9.13	1.90 ± 0.26
SW620-HPLM^{+FBS}	*N = 4*	24.95 ± 0.88	5.14 ± 0.31	60.13 ± 2.48	1.55 ± 0.32
MCF7-RPMI^{+FBS}	*N = 5*	79.31 ± 4.78	30.79 ± 3.95	**180.90 ± 14.03**	5.22 ± 1.01
MCF7-HPLM^{+FBS}	*N = 5*	94.95 ± 2.67	28.85 ± 11.96	**218.51 ± 3.62**	4.74 ± 1.38
A375-RPMI^{+FBS}	*N = 5*	**20.16 ± 0.79**	6.12 ± 0.28	**57.73 ± 9.71**	**3.44 ± 0.90**
A375-HPLM^{+FBS}	*N = 5*	**35.06 ± 3.13**	8.83 ± 1.85	**92.22 ± 6.05**	**2.21 ± 0.51**

**b** cell line - culture media	number of repeats *N* or groups	control ratio *R/E*	control ratio *L/E*	control ratio *L/R*	net ET capacity *E-L* [amol × s^{-1}× x^{-1}]	net *R/E* ratio *(R-L)/E*	*E-L* coupling efficien-cy *(E-L)/E*	*R-L* control efficien-cy *(R-L)/R*	ROX *Rox* / E'_{tot}
SW620-RPMI^{+FBS}	*N = 4*	**0.40 ± 0.01**	0.10 ± 0.01	**0.25 ± 0.03**	56,51± 7.23	**0.30 ± 0.02**	0.90 ± 0.01	**0.75 ± 0.03**	0,03 ± 0.00
SW620-HPLM^{+FBS}	*N = 4*	**0.42 ± 0.02**	0.09 ± 0.01	**0.21 ± 0.01**	54.52 ± 2.26	**0.33 ± 0.01**	0.91 ± 0.01	**0.79 ± 0.01**	0,03 ± 0.01
MCF7-RPMI^{+FBS}	*N = 5*	0.45 ± 0.02	**0.16 ± 0.04**	0.37 ± 0.06	**154.42 ± 16.10**	0.28 ± 0.01	0.84 ± 0.04	0.63 ± 0.06	0,03 ± 0.01
MCF7-HPLM^{+FBS}	*N = 5*	0.43 ± 0.01	**0.13 ± 0.06**	0.31 ± 0.12	**181.91 ± 5.57**	0.30 ± 0.04	0.87 ± 0.06	0.69 ± 0.12	0,02 ± 0.01
A375-RPMI^{+FBS}	*N = 5*	0.35 ± 0.03	0.10 ± 0.00	0.30 ± 0.02	**51.45 ± 9.58**	0.25 ± 0.03	0.90 ± 0.00	0.70 ± 0.02	**0,06 ± 0.00**
A375-HPLM^{+FBS}	*N = 5*	0.37 ± 0.01	0.09 ± 0.01	0.25 ± 0.03	**81.44 ± 5.55**	0.27 ± 0.00	0.91 ± 0.01	0.75 ± 0.03	**0,03 ± 0.01**

cancers MDPI

Table S2. Sunitinib effect on SW620 cell respiration in RPMI^{+FBS} and HPLM^{+FBS}. Median ± IQR (50% range). **(a)** *R*, *L* and *E* corrected for residual oxygen consumption *Rox*. *N* are biological replicates. **(b)** Flux control ratios, net capacities and flux control efficiencies normalized for ROUTINE or ET capacity as an internal normalization to express respiration independent of cell count. Bold font indicates statistical significance at $p \leq 0.05$ using a non-parametric unpaired t-test.

**a** cell line - culture media	condition	number of repeats *N* or groups	ROUTINE *R* [amol×s^{-1}×x^{-1}]	LEAK *L* [amol×s^{-1}×x^{-1}]	ET capacity *E* [amol×s^{-1}×x^{-1}]	*Rox* [amol×s^{-1}×x^{-1}]
SW620-RPMI^{+FBS}	DMSO	*N* = 6	13.11 ± 3.74	2.90 ± 1.47	31.58 ± 7.92	0.76 ± 0.44
	Sunitinib	*N* = 6	13.23 ± 1.42	3.26 ± 0.84	32.23 ± 1.79	0.66 ± 0.40
SW620-HPLM^{+FBS}	DMSO	*N* = 5	9.86 ± 0.45	1.79 ± 0.88	**25.30 ± 5.97**	0.98 ± 0.15
	Sunitinib	*N* = 5	9.59 ± 1.23	2.27 ± 0.74	**19.24 ± 5.22**	0.98 ± 0.27

**b** cell line - culture media	condi-tion	number of repeats *N* or groups	con-trol ratio *R/E*	con-trol ratio *L/E*	con-trol ratio *L/R*	net ET capaci-ty *E-L* [amol×·s^{-1}×x^{-1}]	net *R/E* ratio *(R-L)/E*	*E-L* coupling efficiency *(E-L)/E*	*R-L* control efficien-cy *(R-L)/R*
SW620-RPMI^{+FBS}	DMSO	*N* = 6	0.41 ± 0.02	0.09 ± 0.03	0.22 ± 0.07	28.68 ± 6.69	0.32 ± 0.02	0.91 ± 0.03	0.78 ± 0.07
	Sunitinib	*N* = 6	0.41 ± 0.04	0.10 ± 0.03	0.25 ± 0.04	28.97 ± 2.11	0.31 ± 0.03	0.90 ± 0.03	0.75 ± 0.04
SW620-HPLM^{+FBS}	DMSO	*N* = 5	**0.39 ± 0.07**	**0.07 ± 0.03**	0.18 ± 0.08	**23.51 ± 5.34**	0.32 ± 0.09	**0.93 ± 0.03**	**0.82 ± 0.08**
	Sunitinib	*N* = 5	**0.50 ± 0.07**	**0.12 ± 0.04**	0.24 ± 0.06	**16.97 ± 4.81**	0.34 ± 0.06	**0.88 ± 0.04**	**0.76 ± 0.06**
